# Supplementary material for: Genetic variation for tolerance to the downy mildew pathogen Peronospora variabilis in genetic resources of quinoa (Chenopodium quinoa)
Source: BMC Plant Biol. 2021 Jan 14;21:41. doi: 10.1186/s12870-020-02804-7 (PMC7809748; doi:10.1186/s12870-020-02804-7)
Supplement: Supplementary file 1 — Additional file 1: Figures S1 to S5 and Tables S1 to S3. Supplementary Figures and Tables. [file 12870_2020_2804_MOESM1_ESM.pdf]

Supplementary material 1

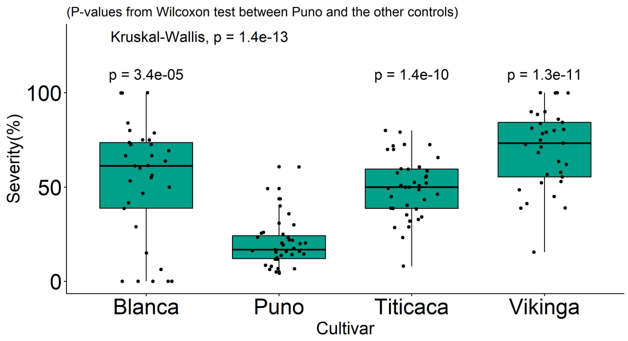

Supplementary Figure S1: Boxplot of the control genotypes from the pilot experiment

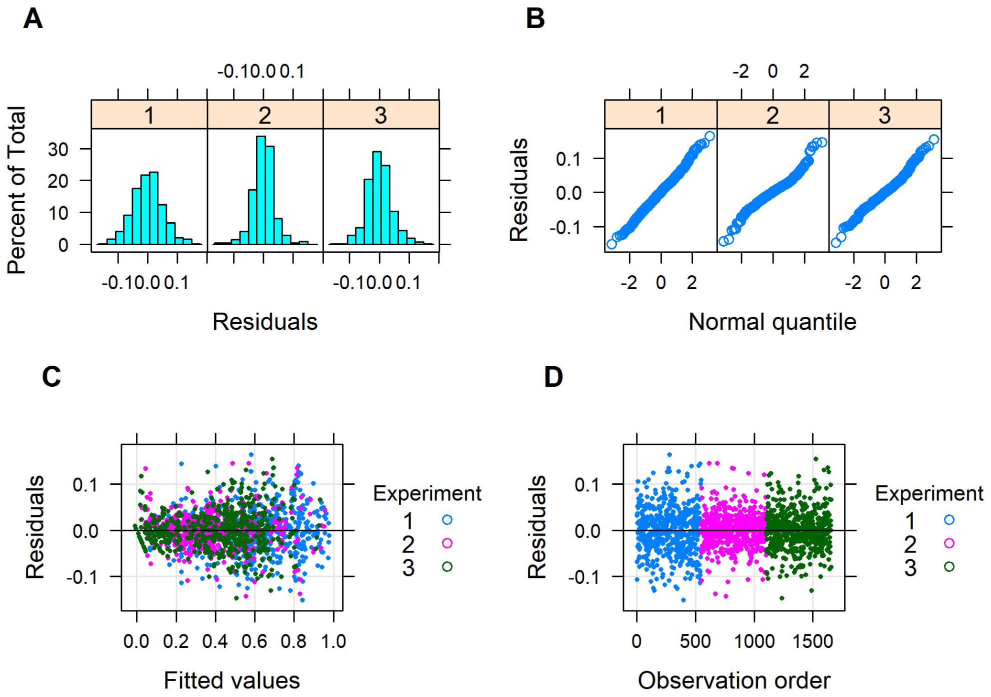

Supplementary Figure S2: Diagnostic plots for a severity LMM with untransformed data and checks included. A) histogram of residuals. B) qq-plot. C) Fitted vs. Residuals plot. D) plot of ordered residuals by experiment.

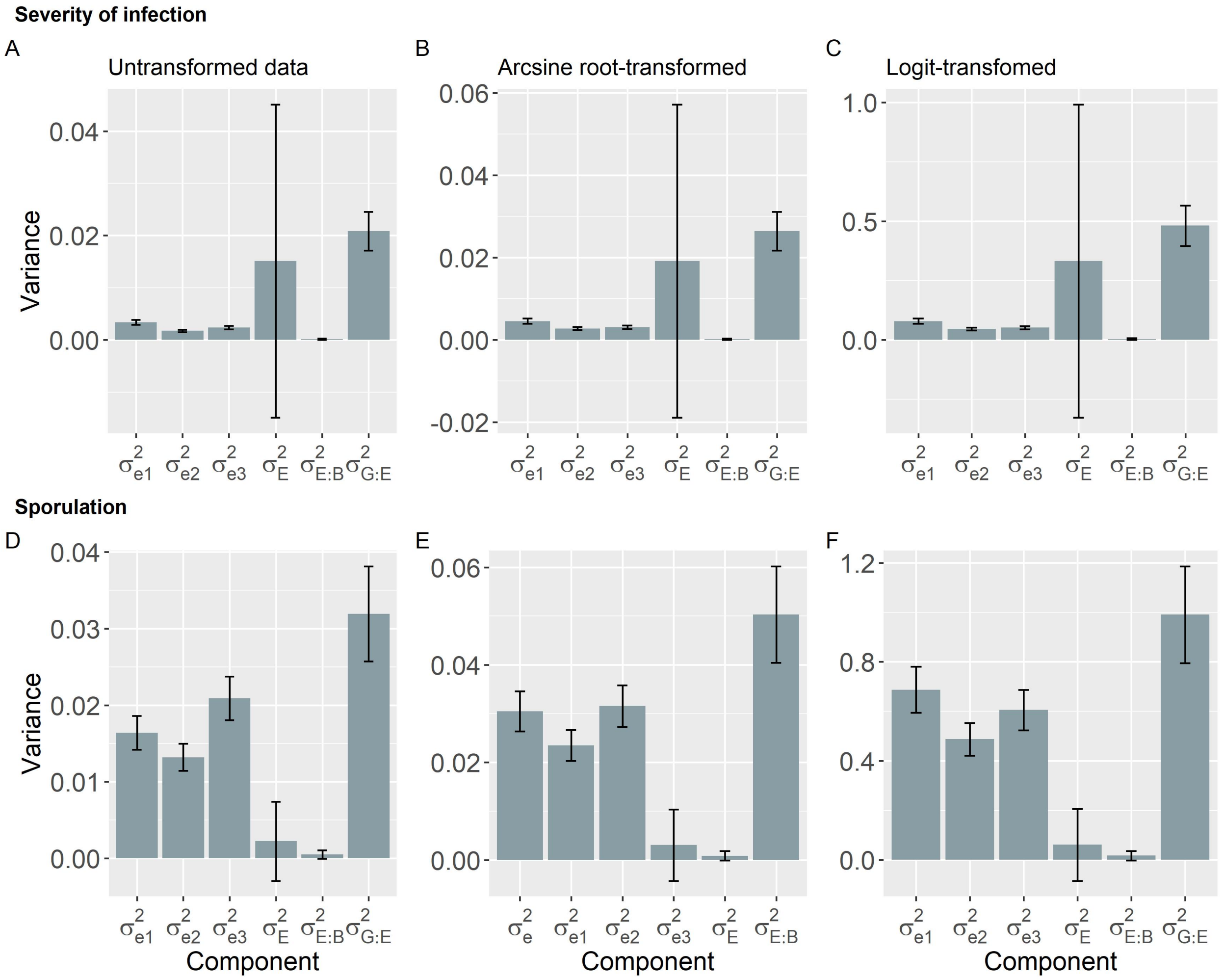

Supplementary Figure S3: Variance components for the trait severity of infection (A-C) and Sporulation (D-F) as estimated with a linear mixed model including the checks. Models were fitted (A,D) without data transformation, (B,E) Arcsine root transformation, and (C,F) logit transformation. σ^2_{e1}, σ^2_{e2}, σ^2_{e3} are residual variances for experiments 1-3; σ^2_{E}; $\sigma^2_{E:B}$ and $\sigma^2_{G:E}$ are variance components for experiments, blocks nested within experiments and the genotype by experiment interaction, respectively.

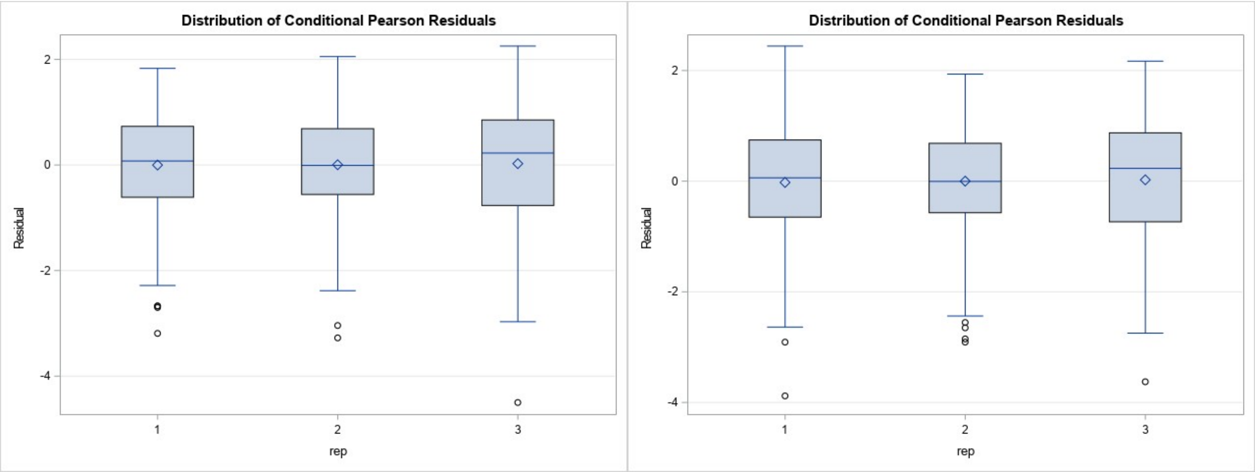

Supplementary Figure S4: Boxplot of the Pearson conditional residuals of the incidence GLMM with (left) and without (right) checks.

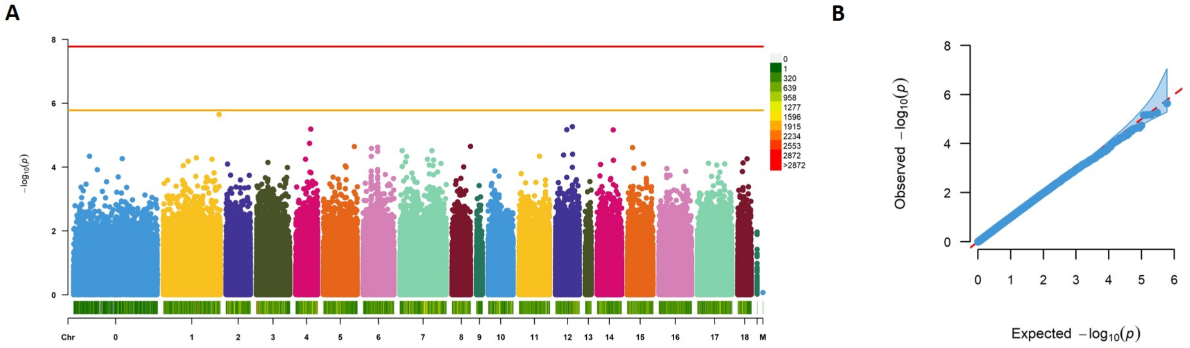

Supplementary Figure S5: Association mapping for downy mildew severity using FarmCPU with models with 3 principal components as covariates. Manhattan plot (A). Red line shows the Bonferroni corrected threshold for $p = 0.01$ and orange line indicates a suggestive threshold (1/number of markers). Bar at the bottom indicates marker density. QQ plot (B) for the FarmCPU model with the 95% confidence interval (light blue); Red line draws the expected distribution of p-values.

Supplementary Table S1: Results of the Restricted Likelihood Ratio Test for the disease severity and sporulation of downy mildew

Variable	*Model*	*Test statistic*	*DF*	*p-value*
Severity	Untransformed - With Checks	44.965	2	$1.72x10^{-10}$
Severity	Untransformed - No Checks	17.119	2	$1.92x10^{-04}$
Severity	Arcsine root - With Checks	29.151	2	$4.68x10^{-07}$
Severity	Arcsine root - No Checks	13.106	2	$1.43x10^{-03}$
Severity	Logit - With Checks	35.056	2	$2.44x10^{-08}$
Severity	Logit - No Checks	23.072	2	$9.77x10^{-06}$
Sporulation	Untransformed - With Checks	22.276	2	$1.45x10^{-05}$
Sporulation	Untransformed - No Checks	6.486	2	$3.90x10^{-02}$
Sporulation	Arcsine root - With Checks	10.646	2	$4.88x10^{-03}$
Sporulation	Arcsine root - No Checks	3.219	2	0.199
Sporulation	Logit - With Checks	12.579	2	$1.86x10^{-03}$
Sporulation	Logit - No Checks	7.307	2	$2.59x10^{-02}$

(Note: each line is a test comparing a null model with homogeneous variances for the error with a model with a heterogeneous variance structure.)

Supplementary Table S2: Estimated variance components for the models fitted for severity and limits for the 95% confidence intervals

**Model**	**Variance Component**	**Estimate**	**95% CI lower limit**	**95% CI upper limit**
Untransformed - No Checks	Experiment:Block	0.0001	-1.14×10^{-05}	2.55×10^{-04}
Untransformed - No Checks	Gen:Exp	0.0225	1.84×10^{-02}	2.66×10^{-02}
Untransformed - No Checks	Exp-1 Error Variance	0.0036	3.14×10^{-03}	4.13×10^{-03}
Untransformed - No Checks	Exp-2 Error Variance	0.0023	1.98×10^{-03}	2.65×10^{-03}
Untransformed - No Checks	Exp-3 Error Variance	0.0032	2.72×10^{-03}	3.62×10^{-03}
Arcsine root - With Checks	Experiment	0.0192	-1.89×10^{-02}	$5.72x10^{-02}$
Arcsine root - With Checks	Experiment:Block	0.0002	-9.63×10^{-06}	3.84×10^{-04}
Arcsine root - With Checks	Gen:Exp	0.0264	2.17×10^{-02}	3.11×10^{-02}
Arcsine root - With Checks	Exp-1 Error Variance	0.0046	3.93×10^{-03}	5.19×10^{-03}
Arcsine root - With Checks	Exp-2 Error Variance	0.0028	2.39×10^{-03}	3.14×10^{-03}
Arcsine root - With Checks	Exp-3 Error Variance	0.0031	2.67×10^{-03}	3.50×10^{-03}
Arcsine root - No Checks	Experiment	0.0239	-2.31×10^{-02}	7.09×10^{-02}
Arcsine root - No Checks	Experiment:Block	0.0002	-1.80×10^{-05}	3.33×10^{-04}
Arcsine root - No Checks	Gen:Exp	0.0276	2.26×10^{-02}	3.27×10^{-02}
Arcsine root - No Checks	Exp-1 Error Variance	0.0047	4.05×10^{-03}	5.35×10^{-03}
Arcsine root - No Checks	Exp-2 Error Variance	0.0034	2.89×10^{-03}	3.85×10^{-03}
Arcsine root - No Checks	Exp-3 Error Variance	0.005	4.29×10^{-03}	5.74×10^{-03}
Logit - With Checks	Experiment	0.3318	-3.28×10^{-01}	9.91×10^{-01}
Logit - With Checks	Experiment:Block	0.0033	-1.64×10^{-04}	6.76×10^{-03}
Logit - With Checks	Gen:Exp	0.4813	3.96×10^{-01}	5.67×10^{-01}
Logit - With Checks	Exp-1 Error Variance	0.0788	6.78×10^{-02}	8.97×10^{-02}
Logit - With Checks	Exp-2 Error Variance	0.0455	3.93×10^{-02}	5.17×10^{-02}
Logit - With Checks	Exp-3 Error Variance	0.0512	4.42×10^{-02}	5.82×10^{-02}
Logit - No Checks	Experiment	0.4876	-4.72×10^{-01}	1.45
Logit - No Checks	Experiment:Block	0.0031	-3.06×10^{-04}	6.46×10^{-03}
Logit - No Checks	Gen:Exp	0.5059	4.13×10^{-01}	5.99×10^{-01}
Logit - No Checks	Exp-1 Error Variance	0.0838	7.20×10^{-02}	9.55×10^{-02}
Logit - No Checks	Exp-2 Error Variance	0.0549	4.71×10^{-02}	6.27×10^{-02}
Logit - No Checks	Exp-3 Error Variance	0.0944	8.07×10^{-02}	1.08×10^{-01}

Supplementary Table S3: Estimated variance components for the models fitted for sporulation and limits for the 95% confidence intervals

Model	Variance Component	Estimate	95% CI lower limit	95% CI upper limit
Untransformed - With Checks	Experiment	0.0022	-2.92×10^{-03}	7.38×10^{-03}
Untransformed - With Checks	Experiment:Block	0.0005	-6.68×10^{-05}	1.03×10^{-03}
Untransformed - With Checks	Gen:Exp	0.0319	2.57×10^{-02}	3.82×10^{-02}
Untransformed - With Checks	Exp-1 Error Variance	0.0164	1.42×10^{-02}	1.86×10^{-02}
Untransformed - With Checks	Exp-2 Error Variance	0.0132	1.14×10^{-02}	1.50×10^{-02}
Untransformed - With Checks	Exp-3 Error Variance	0.0209	1.81×10^{-02}	2.38×10^{-02}
Untransformed - No Checks	Experiment	0.0093	-9.51×10^{-03}	2.81×10^{-02}
Untransformed - No Checks	Experiment:Block	0.0005	-8.63×10^{-05}	1.01×10^{-03}
Untransformed - No Checks	Gen:Exp	0.0365	2.90×10^{-02}	4.40×10^{-02}
Untransformed - No Checks	Exp-1 Error Variance	0.0188	1.62×10^{-02}	2.14×10^{-02}
Untransformed - No Checks	Exp-2 Error Variance	0.0166	1.43×10^{-02}	1.90×10^{-02}
Untransformed - No Checks	Exp-3 Error Variance	0.0216	1.87×10^{-02}	2.46×10^{-02}
Arcsine root - With Checks	Experiment	0.0031	-4.24×10^{-03}	1.04×10^{-02}
Arcsine root - With Checks	Experiment:Block	0.0009	-1.13×10^{-04}	1.84×10^{-03}
Arcsine root - With Checks	Gen:Exp	0.0503	4.05×10^{-02}	6.02×10^{-02}
Arcsine root - With Checks	Exp-1 Error Variance	0.0305	2.64×10^{-02}	3.46×10^{-02}
Arcsine root - With Checks	Exp-2 Error Variance	0.0235	2.03×10^{-02}	2.67×10^{-02}
Arcsine root - With Checks	Exp-3 Error Variance	0.0316	2.73×10^{-02}	3.58×10^{-02}
Arcsine root - No Checks	Experiment	0.0094	-1.00×10^{-02}	2.89×10^{-02}
Arcsine root - No Checks	Experiment:Block	0.0009	-1.41×10^{-04}	1.87×10^{-03}
Arcsine root - No Checks	Gen:Exp	0.0542	4.31×10^{-02}	6.53×10^{-02}
Arcsine root - No Checks	Error Variance	0.031	2.86×10^{-02}	$3.34x10^{-02}$
Logit - With Checks	Experiment	0.0612	-8.42×10^{-02}	0.207
Logit - With Checks	Experiment:Block	0.0168	-2.48×10^{-03}	3.60×10^{-02}
Logit - With Checks	Gen:Exp	0.9906	0.795	1.19
Logit - With Checks	Exp-1 Error Variance	0.6876	0.595	0.780
Logit - With Checks	Exp-2 Error Variance	0.4873	0.422	0.553
Logit - With Checks	Exp-3 Error Variance	0.6055	0.523	0.688
Logit - No Checks	Experiment	0.1829	-0.195	0.561
Logit - No Checks	Experiment:Block	0.0172	-2.83×10^{-03}	3.73×10^{-02}
Logit - No Checks	Gen:Exp	1.0652	0.846	1.28
Logit - No Checks	Exp-1 Error Variance	0.7415	0.64	0.843
Logit - No Checks	Exp-2 Error Variance	0.5657	0.487	0.644
Logit - No Checks	Exp-3 Error Variance	0.6145	0.531	0.698
