# Supplementary material for: Genetic variation for tolerance to the downy mildew pathogen Peronospora variabilis in genetic resources of quinoa (Chenopodium quinoa)
Source: BMC Plant Biol. 2021 Jan 14;21:41. doi: 10.1186/s12870-020-02804-7 (PMC7809748; doi:10.1186/s12870-020-02804-7)
Supplement: Supplementary file 3 — Additional file 3: Supplementary File 2. Detailed protocol for the isolation and maintenance of the downy mildew pathogen Peronospora variabilis. [file 12870_2020_2804_MOESM3_ESM.pdf]

Protocols of the experimental design

- List of biological control agents used in this study

Table 1: Species used to control pests

Species
Aphidius Col/Erv/Aphel
Macrolophus caliginosus
Amblyseius cucumeris
Aphidoletes aphidimyza
Orius majusculus/laevigatus
Encarsia Formosa
Hypoaspis Miles
Amblyseius swirskii
Amblyseius Californicus
Chrysoperla carnea

- Protocol for propagation of the *Peronospora variabilis* isolate
  - Rinse infected leaves with distilled sterile water.
  - Filter the solution and re-suspend the spores in sterile water.
  - Calibrate the spore's concentration to 10^5 spores per ml; give the solution a cold snap for 5 minutes; load the bottle feeder of an air brush.
  - Set the pressure to 50 bar and spray 7 to 8 weeks old potted quinoa plants of the Danish cultivar Vikinga and Bolivian cultivar Blanca, making sure to cover all the leaves
  - Cover inoculated plants with polyethylene bags and keep them in a dark chamber at 22degree C during the day and 15degree C during the night for 48 hours.
  - Remove the bags and keep the plants under a 16h photoperiod and a 22degree C/18degree C day/night temperature cycle for five days, until the first signs of infection are visible.
  - Cover the plants with polyethylene bags again, reduce nighttime temperature to 15degree C, and keep them in a dark chamber for 24 hours. At this point, there should be signs of sporulation on the abaxial side of the leaves.
  - Collect leaves with signs of sporulation and prepare a spore solution.
  - Repeat this process as necessary.
